# Projecting the economic burden of type 1 and type 2 diabetes mellitus in Germany from 2010 until 2040

**DOI:** 10.1186/s12963-024-00337-x

**Published:** 2024-07-18

**Authors:** Dina Voeltz, Maximilian Vetterer, Esther Seidel-Jacobs, Ralph Brinks, Thaddäus Tönnies, Annika Hoyer

**Affiliations:** 1 Biostatistics and Medical Biometry, Medical School OWL, Bielefeld University, Universitätsstr. 25, 33615 Bielefeld, Germany; 2 Department of Statistics, Ludwig-Maximilians-University, Munich, Germany; 3 Institute for Biometrics and Epidemiology, German Diabetes Center, Leibniz Institute for Diabetes Research at Heinrich-Heine-University, Düsseldorf, Germany; 4 German Center for Diabetes Research, Partner Düsseldorf, München-Neuherberg, Germany; 5 Chair for Medical Biometry and Epidemiology, Faculty of Health/School of Medicine, Witten/Herdecke University, Witten, Germany

**Keywords:** Cost analysis, Economic burden of disease, Epidemiology, Healthcare costs, Projection, Type 1 diabetes, Type 2 diabetes

## Abstract

**Background:**

The aim is to estimate age- and sex-specific direct medical costs related to diagnosed type 1 and type 2 diabetes in Germany between 2010 and 2040.

**Methods:**

Based on nationwide representative epidemiological routine data from 2010 from the statutory health insurance in Germany (almost 80% of the population’s insurance) we projected age- and sex-specific healthcare expenses for type 1 and 2 diabetes considering future demographic, disease-specific and cost trends. We combine per capita healthcare cost data (obtained from aggregated claims data from an almost 7% random sample of all German people with statutory health insurance) together with the demographic structure of the German population (obtained from the Federal Statictical Office), diabetes prevalence, incidence and mortality. Direct per capita costs, total annual costs, cost ratios for people with versus without diabetes and attributable costs were estimated. The source code for running the analysis is publicly available in the open-access repository Zenodo.

**Results:**

In 2010, total healthcare costs amounted to more than €1 billion for type 1 and €28 billion for type 2 diabetes. Depending on the scenario, total annual expenses were projected to rise remarkably until 2040 compared to 2010, by 1–281% for type 1 (€1 to €4 billion) and by 8–364% for type 2 diabetes (€30 to €131 billion). In a relatively probable scenario total costs amount to about €2 and €79 billion for type 1 and type 2 diabetes in 2040, respectively. Depending on annual cost growth (1% p.a. as realistic scenario vs. 5% p.a. as very extreme setting), we estimated annual per capita costs of €6,581 to €12,057 for type 1 and €5,245 to €8,999 for type 2 diabetes in 2040.

**Conclusions:**

Diabetes imposes a large economic burden on Germany which is projected to increase substantially until 2040. Temporal trends in the incidence and cost growth are main drivers of this increase. This highlight the need for urgent action to prepare for the potential development and mitigate its consequences.

**Supplementary Information:**

The online version contains supplementary material available at 10.1186/s12963-024-00337-x.

## Introduction

Worldwide, the number of people with diabetes and its associated costs have increased considerably [1–4]. In 2021, the global diabetes prevalence was estimated 10.5% among adults (20–79 years), i.e. 537 million people, and caused healthcare costs of at least $966 billion [3]. The absolute global economic burden is projected to grow to more than $2 trillion by 2030 [5]. In Germany, at least 7% of the population have diabetes, while this number is predicted to increase by up to 77% over the next 20 years [6]. This makes it a major health concern and challenge with regards to medical and economic resource planning. However, little is known about diabetes-related healthcare expenditures in Germany [7]. Cost of illness estimates are relevant from economic, medical and political point of view, and are necessary for effective healthcare management, meeting future medical needs and evaluating measures for prevention and intervention.

The only two available diabetes-related cost projections for Germany estimated an increase by 79% between 2010 and 2040 (from about €12 billion to €21 billion) [7], or healthcare expenditures of $30 to $56 billion in 2030 [2]. However, the latter forecast lacks detailed country-specific input data, uses oversimplified methods and is based on a debatable assumption of constant diabetes prevalence over time. This also applies to international cost studies which are inherently limited by the methods applied [7]. For instance, they do not include an analysis of cost data or future cost trends such as inflation, ignore demographic changes, are limited to one sex, certain ages or other demographic input factors, do not differentiate the types of diabetes, or disregard the interplay of prevalence, incidence and mortality [6–9]. However, with regards to diabetes, the incidence and mortality majorly impact disease dynamics and ignorance leads to severe underestimation of future prevalence, which is one of the input factors for cost analyses [6, 8, 10]. Besides, the diabetes types are considerably different in their clinical representation, onset and progression [10]. Demographic shifts such as the aging population is relevant for type 2 diabetes, as its prevalence peaks amongst the cost-intensive older age groups [7]. Consequently, accurate projection methods of future chronic disease-specific costs need to be capable of reflecting the complex interplay simultaneously and differentiate where appropriate.

In this study, we use nationally representative data and a comprehensive and transparent method to project the sex-, age-, year- and diabetes-type specific per capita costs, total excess costs and cost ratios of people with and without type 1 and type 2 diabetes, as well as attributable costs in Germany from 2010 until 2040.

## Methods

### Data

We used the official population projection of the German Federal Statistical Office (FSO) [11], aggregated and published data on the incidence, prevalence and mortality of individuals with versus without type 1 and 2 diabetes [6, 8–10, 12, 13], as well as information on healthcare expenditures of the general German population (Additional Table 1 in Additional file 1) [14].

#### Demographic inputs

We obtained the expected age- and sex-specific population distribution and the mortality of the general population in Germany for each year from 2010 to 2040 and all ages from 0 to 100 years from the FSO [11]. Six variants that represent rather realistic future developments instead of extreme assumptions [11] were considered to account for changes in migration, birth rates and population ageing (Additional Table 2 in Additional file 1). We used variant 2 (G2L2W2) as baseline, which assumes a birth rate (G2) of 1.55 children per woman, a life expectancy (L2) at birth in 2040 of about 84 and 88 years for men and women, respectively, and a long-term net migration (W2) of annually 290,000 people. These assumptions align relatively well with values observed in Germany in 2020 and 2021 which include dynamics such as the COVID-19 pandemic or the Russo-Ukrainian war [15].

#### Disease-specific epidemiological inputs

Initial prevalence and incidence information were obtained from claims data that are considered representative for Germany and that were featured in recent projections of diabetes prevalence [6, 8–10]. The data comprise information on the age and sex of 65 million insures in 2010 (about 80% of the total population) from all German statutory health insurances (SHI) and their diabetes diagnoses. The data were interpolated with natural splines to obtain information for each age between 0 and 100 years. We defined type 1 and type 2 diabetes based on the International Classification of Diseases-10 (ICD-10) codes E10–E14. In 2010, about 7% of the German population were diagnosed with type 2 and 0.3% with type 1 diabetes. Regarding the age- and sex-specific mortality rate ratio (MRR) of people with versus without diabetes, we aligned with previous studies investigating diabetes in Germany [8, 10] and included nationally representative MRR estimates of type 2 diabetes from 2014 provided by Schmidt et al. [13] and estimates from Carstensen et al. [12, 16] to approximate the MRR of type 1 diabetes in Germany [10].

#### Cost inputs

The average per capita healthcare costs for people with and without diabetes were obtained from aggregated claims data from an almost 7% random sample of all German people with SHI [14], i.e., of almost 80% of the German population. The expenses include direct per capita costs for physicians, dentists, pharmacies, hospitals, sick benefits and others in 2010 in Germany from payer perspective. The included ICD-10 codes allowed to differentiate between costs related to type 1 or 2 diabetes. To quantify the medical costs related to type 1 and 2 diabetes in 2010, we first sorted the data by expenditure field (physicians, dentists, pharmacies, hospitals, sick benefits and others), diagnosis (type 1, type 2 or no diabetes), sex (men, women), and age group (22 groups of 5-year intervals). Other healthcare expenses contain, among others, therapeutic remedies and aids, expenses for services abroad, inpatient benefits and rehabilitation service. The data were linearly interpolated (using the approx()-function in R) between age groups to obtain data for all ages from 0 to 100 years. Next, we computed the sum of all costs spend for any of the six expenditure fields across all ages differentiated by sex and by diagnosis (i.e., type 1, type 2 or no diabetes). The total annual costs were then divided by the number of people with type 1, type 2 or no diabetes, respectively, to obtain annual average per capita costs by sex and diagnosis. These data serve as starting values of per capita costs, cost ratios for people with versus without diabetes and attributable costs in 2010. Due to regulations on data protection, routine SHI data were provided in an anonymous and aggregated form (§ 5 Data Transparency Regulation, paragraph 4).

### Projection model

As measures of interest, we modelled direct total and excess costs, cost ratios for people with versus without diabetes, and population attributable costs (see Additional Table 1 in Additional file 1) using the statistical software R, version 4.1.2 (R Foundation for Statistical Computing). The source code is published in the open-access repository Zenodo [17].

All outcome measures are differentiated by year, sex and diabetes type. Further, all analyses comprise information on all ages between 0 and 100 years. Although type 1 diabetes is most prevalent in children and adolescents and type 2 more common among older adults, the types of diabetes are not limited to the respective age groups and can occur at any age. Therefore, for consistency, to enable comparison between diabetes types and to consolidate the diabetes surveillance, we include data on type 1 diabetes in adults and type 2 diabetes in children and adolescents.

#### Epidemiological development

We first estimated the observed sex-specific prevalence of diagnosed type 1 and 2 diabetes in Germany in 2010 for all ages between 0 and 100 years. Second, we used a partial differential equation (PDE) that originates in the illness–death model (IDM) to project the age- and sex -specific prevalences until 2040 [6, 8, 10, 18]. Our PDE describes the relation between prevalence, incidence and mortality as a function of age and calendar time, and thereby allows to incorporate future trends in the incidence and mortality. Applying the projected prevalence to projected population counts yielded the number of individuals with type 1 or type 2 diabetes in Germany for each year between 2010 and 2040.

#### Cost projection

Using the aggregated cost data, we computed the total costs for each age and sex stratum by multiplying the number of people with or without diabetes and the respective per capita costs in 2010. We added these costs across all strata to obtain the total costs depending on diabetes status, i.e., diagnosed type 1, type 2 or no diabetes. We obtained the average per capita costs in 2010 by dividing the total costs and the age- and sex-specific number of people with or without diabetes. We stratified the average per capita costs by sex and diabetes status and interpolated between the age groups to calculate average per capita costs for all ages from 0 to 100 years. Diabetes-related excess costs were defined as costs of a person with type 1 or 2 diabetes that go beyond the costs of people without diabetes. To project the excess costs until 2040, we multiplied the excess costs with a mean annual growth rate of 0%, 1% or 5% depending on the respective scenario. The mean annual growth rate denotes the average annual percentage change in excess costs related to diabetes over the overall projection horizon (for details see Additional Table 1 in the Additional file).

Total diabetes-related future costs from 2010 to 2040 were calculated as sum of the product of the projected population sizes, projected prevalence and projected average per capita costs for each year. Total annual excess costs are defined analogously, but include average per capita excess costs instead of average per capita costs. Using the projected cost data, we computed annual age- and sex-specific cost ratios (*R*) for people with type 1 or 2 diabetes relative to people without. The age- (*a*), sex- (*s*), diabetes type- (*d*) and time-specific (*t*) attributable costs (*PAC*) were defined as

$$\:{PAC}_{asdt}=\:\frac{{p}_{asdt}\times\:({R}_{asdt}-1)}{1+{p}_{asdt}\times\:({R}_{asdt}-1)}$$

with *p* denoting the prevalence of the respective diabetes type [14]. To extrapolate from the data of the random sample (6.8%) o the whole German population, we divided the sum of the total costs by the sample size. The resulting year-specific quotient was multiplied with the respective population attributable costs for each year.

#### Scenario analyses

To account for uncertainty, we modelled several demographic-, diabetes- and cost-related future dynamics. We constructed 16 scenarios motivated by previous papers from Waldeyer et al. [7], Tönnies et al. [6] and Voeltz et al. [8, 10], who projected diabetes-related cost and prevalence in Germany for similar time horizons (details given in Table S2). Scenario 1 represents a base-case scenario. It assumes moderate demographic developments (variant G2L2W2) and is limited to an annual 2% decrease in the MRR with no changes made to any other cost or epidemiological input. Scenarios 2 to 8 account for potential epidemiological developments, scenarios 9 and 10 model the robustness of our results concerning changes in diabetes-related costs and scenarios 11 to 15 assess hypothetical demographic trends. Scenario 16 assumes moderate demographic development, a mean annual cost growth of 1% [7], an annual 1% increase in the incidence [6, 8, 10] and a decrease of 2% in the MRR [6, 8, 10]. We consider this scenario to be the most probable one as it models future trends relatively conservatively and is based on previous findings and on values from other diabetes-related projections [6, 7, 10].

#### Sensitivity analyses

We reflect on potential error in the input values and future trends of the model parameters using the relatively extreme scenarios 6, 7, 8, and 10 which return upper and lower projection bounds.

## Results

### Per capita costs

**Fig. 1:**
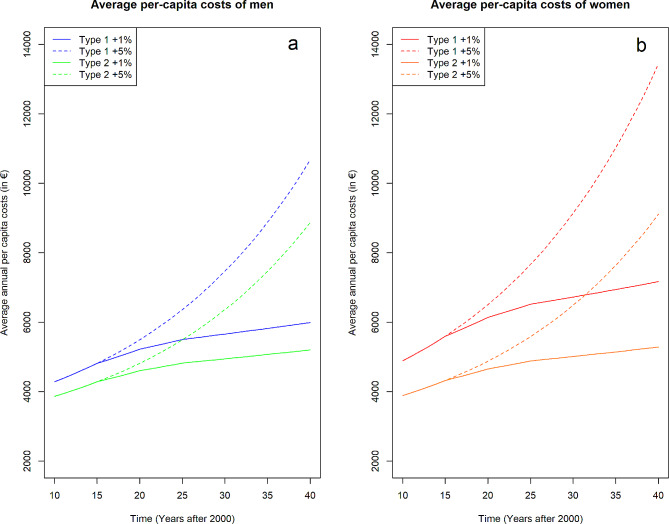
Projected average per capita costs Projected average per capita costs (in €) of people with type 1 or 2 diabetes in the statutory health insurance in Germany between 2010 and 2040 (stratified by sex) assuming annual cost growth rates of 1% or 5%. Panel **a** shows average per-capita costs of men with type 1 or 2 diabetes, panel **b** displays the projected per-capita costs for women, respectively

In the reference year 2010, average per capita healthcare expenses of people insured in the SHI in Germany amounted to €4,285 for men and €4,889 for women with type 1 diabetes, and €3,868 for men and €3,889 for women with diagnosed type 2 diabetes. Per capita costs for people without diabetes amounted to 2,360 and €2,316 for men and women, respectively. Assuming a moderate cost growth of 1% per year as in scenario 9 and 16, annual per capita costs reached on average €6,581 for type 1 and €5,245 for type 2 diabetes in 2040 (Fig. 1 and Additional Fig. 1 in Additional file 1). Accordingly, the increase was markedly higher when assuming an annual cost growth rate of 5% (scenario 10) to approximately €12,057 for type 1 and €8,999 for type 2 diabetes.

### Total annual healthcare costs

**Fig. 2:**
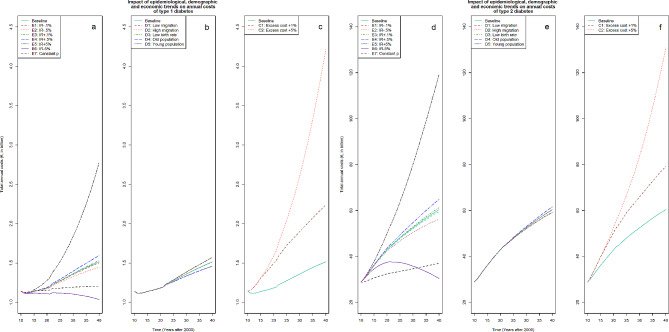
Projected annual total healthcare expenses Projected annual total healthcare expenses for people with diagnosed diabetes type 1 or type 2 from 2010 until 2040 in Germany for different epidemiological, demographic or cost development scenarios. Results for type 1 diabetes are shown in panel **a** (epidemiological trends), **b** (demographic trends) and **c** (economic trends), while panel **d** (epidemiological trends), **e** (demographic trends) and **f** (economic trends) display results for type 2 diabetes

All scenarios with only one exception projected rising total annual healthcare expenses over time (Additional Tables 3 and 4 in Additional file 1). In 2010, the observed total annual costs in Germany amounted to about €1 and almost €29 billion for type 1 and type 2 diabetes, respectively. In 2040 and depending on the scenario, costs were projected to exceed this by 1–281% for type 1 and by 8–364% for type 2 diabetes (Fig. 2). Our baseline scenario 1 resulted in total annual costs of about €1.5 billion for type 1 (+ 30%) and more than €60 billion for type 2 diabetes in 2040. The most probable scenario 16 returned total costs of about €2 and €79 billion related to type 1 or type 2 diabetes in 2040, respectively.

### Cost ratio

**Fig. 3:**
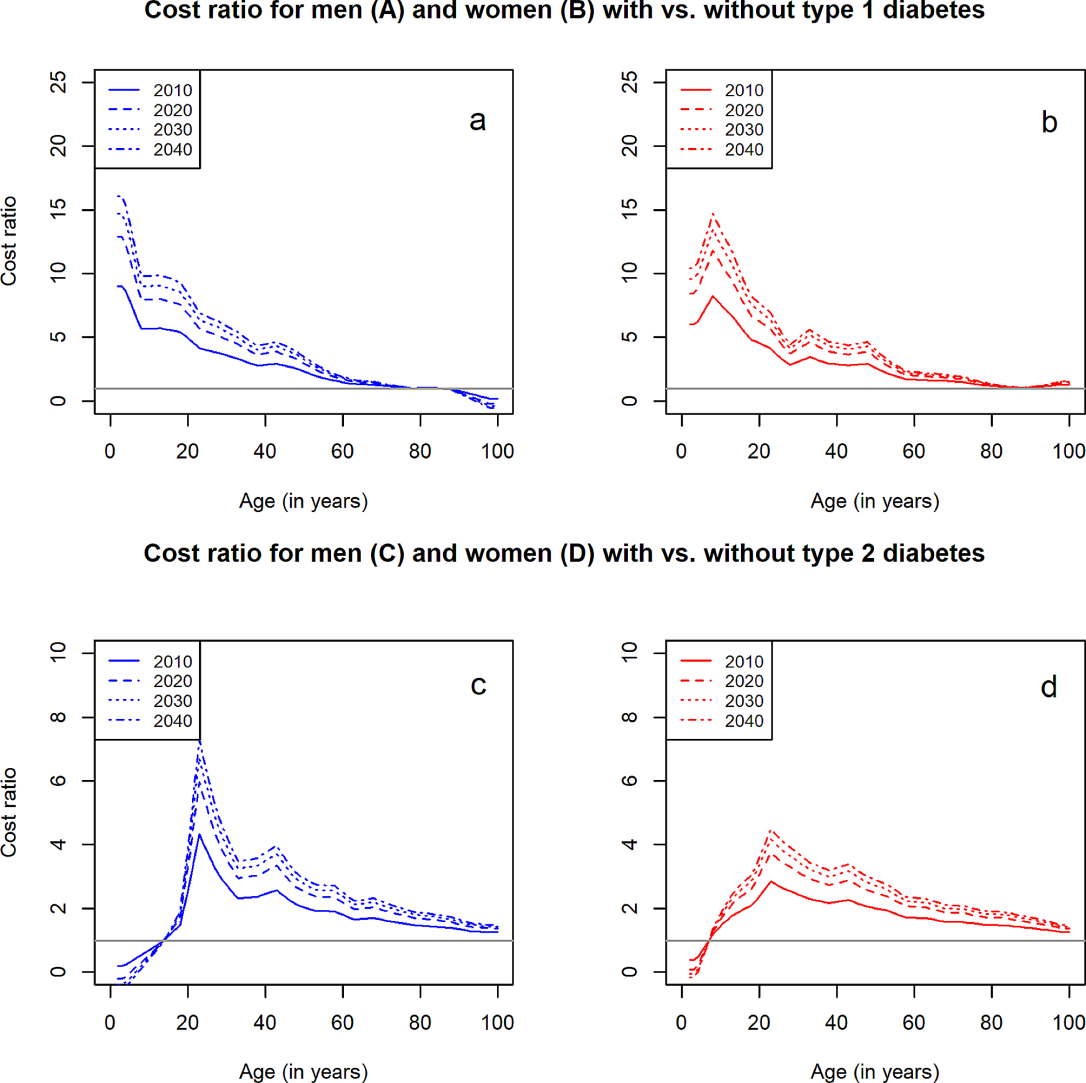
Projected age-specific cost ratios of the total healthcare expenses Age-specific cost ratios of the total healthcare expenses (in €) between men and women with diagnosed type 1 or 2 diabetes versus men and women without in the statutory health insurance in Germany in 2010, 2020, 2030 and 2040. Panel **a** and **b** show results for men and women with type 1 diabetes, panel **c** and **d** display the projected cost ratios for men and women with type 2 diabetes, respectively

Most likely (scenario 16), annual healthcare expenses in 2040 will be 2.8- and 3.2-fold higher for men and women with type 1, and 3.8- and 3.3-fold higher for men and women with type 2 diabetes compared to an insured person without diabetes, respectively (Fig. 3 and Additional Fig. 2 in Additional file 1). Generally, cost ratios were largest among younger ages and decreased with increasing age. For people with type 1 diabetes aged between 0 and 10 years, the discrepancy is highest, results showed 7-fold higher costs in 2010 and up to 15-fold higher costs in 2040. Regarding type 2 diabetes, the cost imbalance between people with versus without diabetes is less high and peaks around the age of 20 to 30 years (about 3- to 4-fold higher in 2010 and 6-fold higher in 2040).

### Excess costs

In 2010, diabetes caused total medical excess costs of €0.3 billion for men and €0.3 billion for women with type 1 diabetes, and almost €6 billion for men and €5 billion for women with type 2 diabetes (Additional Tables 3 and 4 in Additional file 1). Our base-case scenario predicted an increase to a total of €0.8 billion for type 1 and €21 billion for type 2 diabetes in 2040, while scenario 16 projected excess costs of €1.5 and €40 billion, respectively.

### Population attributable costs and cost extrapolation

**Fig. 4:**
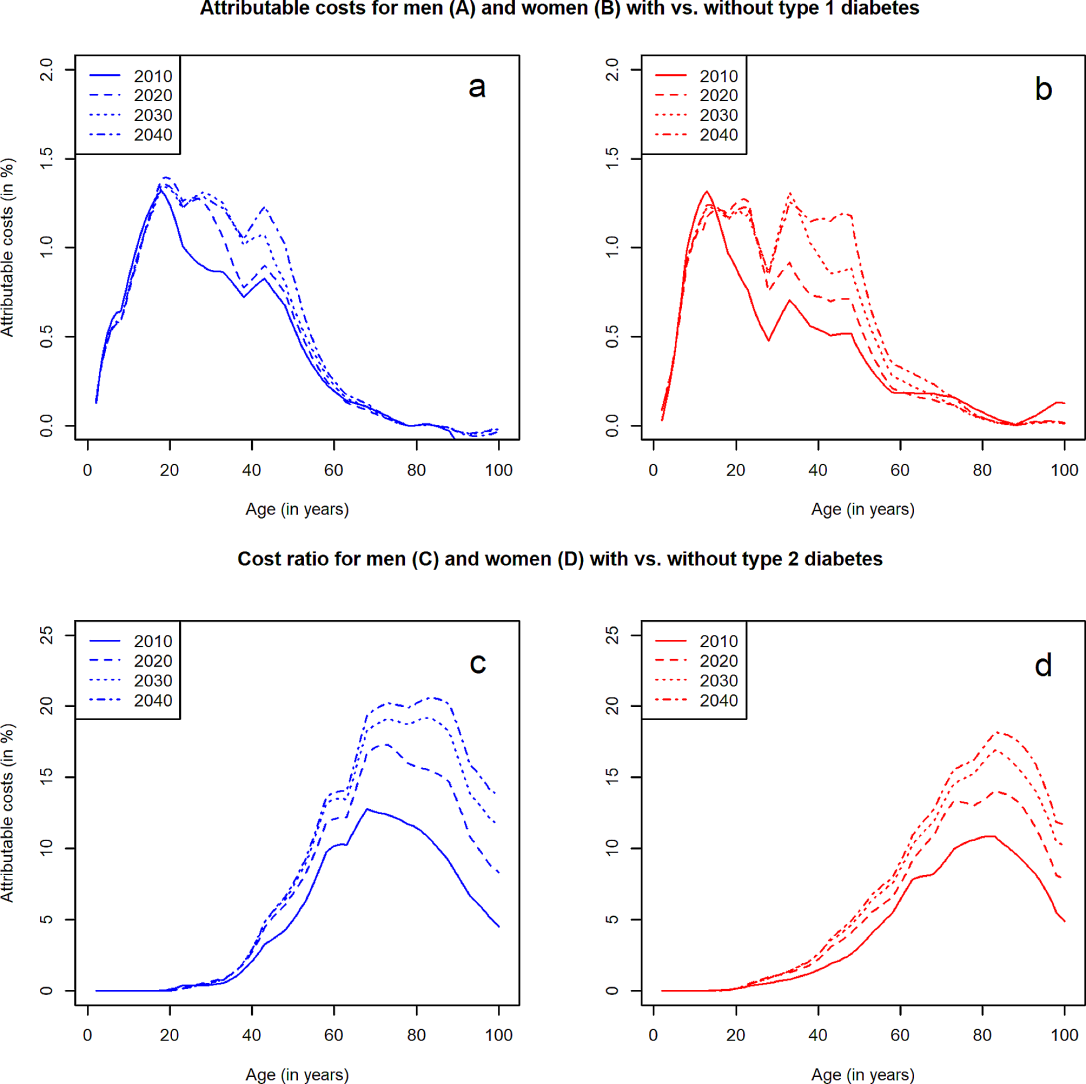
Projected age- and sex-specific attributable costs Age- and sex-specific attributable costs (%) of diagnosed type 1 or 2 diabetes in the statutory health insurance in Germany in 2010, 2020, 2030 and 2040. Panel **a** and **b** show results for men and women with type 1 diabetes, panel **c** and **d** display the projected attributable costs for men and women with type 2 diabetes, respectively

In Germany in 2010, SHI expenses amounted to ~€160 billion [14]. On average, 3.8% and 10.2% thereof are attributable to the medical care of type 1 and type 2 diabetes, respectively. For the most probable scenario 16 in 2040, attributable costs of type 1 diabetes will be almost 8%, and about 26% for type 2 diabetes. Extrapolating from our sample to the whole population showed that this corresponds to total direct costs of €0.55 and more than €14 billion for type 1 and type 2 diabetes in 2010 and about €1 and €37 billion in 2040, respectively. Attributable costs of type 2 versus type 1 diabetes are markedly higher (Fig. 4). For type 1 diabetes, attributable costs are highest among younger ages. Vice versa, for type 2, these increase with increasing age. We projected slightly higher attributable costs for men compared to women with type 2 diabetes.

## Discussion

### Principal findings

In Germany in 2010, direct total annual healthcare costs amounted to more than €1 billion for type 1 and €28 billion for type 2 diabetes. Expenditures of SHI for diabetes are projected to rise remarkably until 2040, to about €1 to €4 billion and €30 to €131 billion, respectively. In a most probable scenario total costs are projected to increase to about €2 and €79 billion related to type 1 or type 2 diabetes in 2040, respectively. Previous findings showed that future prevalence will increase strongest in the costliest age groups for the corresponding diabetes type (20 to 40 years for type 1 and 60 + years for type 2 diabetes) [6, 10]. Further, our scenarios show that future costs are highly sensitive to and are mainly driven by assumptions in the excess costs. Demographic changes such as population ageing had little influence on the cost projection. The combination of these dynamics might explain a large part of the cost growth.

### Scenario comparison

Forecasting is fraught with uncertainty as unexpected cultural, political, economic or medical shifts may prompt change and current trends develop differently than assumed. Therefore, we assessed several scenarios and can only speculate which scenario is most likely. Comparing all scenarios (Fig. 2) showed that the cost growth is mainly attributable to rising incidence and cost rates, with little impact of population ageing.

Generally, demographic changes had little influence on the projection. With regards to migration, Germany reports a positive and rising migration balance since several decades making scenario 12 (assuming high migration) likelier than scenario 11 (low migration) [15]. Population ageing, analysed in scenario 14 versus 15, is related to an increased number of high-risk individuals and an increased life expectancy of people with diabetes and therefore, contributes to a higher number of diseased people. The combination of population ageing and per capita costs that increase with age might explain the observed minor impact on future cost growth.

The projected economic burden was highly sensitive to future cost growth rates. If direct medical per capita excess costs would inflate by 1% annually (scenario 9), the total costs of type and type 2 diabetes in Germany would increase by 100% for type 1 and 180% for type 2 diabetes. By contrast, assuming a very extreme (and rather unrealistic) cost growth of 5% (scenario 10), total annual costs in 2040 were projected to increase by 281% and 364%, respectively. Bommer et al. [5] projected global total costs of diabetes in adults (aged 20–79 years) through the year 2030. Their projection led to cost increases in the total absolute costs related to diabetes until 2030 compared to 2015 by 61–88%. Waldeyer et al. [7] project increases from 27% to up to 146% (depending on the scenario) from 2010 to 2040 in the total healthcare costs of all individuals with diabetes in Germany. In light of the mentioned findings, our estimates seem relatively plausible except for the extreme scenario 10. However and as acknowledged, forecasting is always fraught with uncertainty and scenario 10 is intentionally set as relatively extreme setting to provide an upper bound of future costs.

Another large part of the rising costs is possibly explained by the increase in prevalence attributable to future disease-specific dynamics [6, 8, 10]. Scenario 8 assumes that the age-and sex-specific prevalence remains constant as in 2010 and results in an increase of total costs in 2040 compared to 2010 of 1% and 29% for type 1 and 2 diabetes. Though, this probably severely underestimates the actual disease and cost burden [6, 8, 10]. More realistically are changing incidence rates (as in scenario 2 to 5), which resulted in increased annual costs by 28–41% for type 1 and by 109–128% for type 2 diabetes. Scenario 6 and 7, assuming a 5% annual increase or decrease in the incidence, account for extreme events such as the impact of the SARS-CoV-2 pandemic, which is associated with an increased risk of new-onset diabetes [8, 10]. In 2040 compared to 2010, these scenarios lead to changes of the total cost by -1% or 149% for type 1 and by 6% or 322% for type 2 diabetes. This variability highlights the consequences of future incidence trends on upcoming healthcare expenditures.

### Strengths and weaknesses

While our analysis provides important information for chronic-disease related research, future diabetes healthcare and policy interventions, it does have limitations.

Using mostly nationwide data, the risk of bias is considerably low. Though, one weakness of our data arises with the time that elapsed since the data collection in 2010. The current situation might differ and profound and unexpected shifts, such as the COVID-pandemic or the Russo-Ukrainian War, lead to discrepancy between current and past trends.

Further, our analysis shares the drawbacks of all studies that encounter SHI data: For instance, not all SHI funds operate nationwide as well as SHI funds differ in the quantity and composition of their members (e.g., socio-economic status) [19, 20]. Ultimately, this may result in biased results, it may reduce the comparability of the studied population across different SHI or may impede the generalisability to the whole German population [20]. As an illustration, Heidemann et al. [19] have shown that the diabetes prevalence is higher among persons insured by SHI compared to people with private health insurance. However, our study can be considered representative for the people in SHI. Only those people in Germany with private insurance (the remaining 10% of the population) may have different social status and risk profiles.

In addition to direct medical costs, diabetes is associated with indirect social and productivity costs such as premature mortality, disability, and higher rates of lost work time [7]. It is beyond the scope of our study because precise information on the associated indirect costs is limited. The estimation of starting values for a projection requires additional methods and assumptions, for instance age restrictions because indirect costs only accrue from productivity losses arising from diabetes in working-age people [21]. Resulting, uncertain estimates would render any projection intrinsically highly speculative. We recommend future research to investigate this knowledge gap.

Another limitation of our study is that costs of diabetes not only differ by sex and age but also comorbidities [22] which was not explicitly considered in this study. It may be interesting to investigate this issue in further detail in future studies.

Further, basing our analysis on aggregated data (i.e. aggregated to population level by year, sex and specific age groups) may have led to minor imprecision since we had to interpolate between the age groups to derive data for all ages between 0 and 100 years. Note that this limitation holds for the aggregated cost and epidemiological input data. However, to project epidemiological scenarios aggregated data was sufficient, as the discussed PDE works fine on such data. Demographic input information was publicly available for all years, both sex and all ages, hence, no issues aroused.

In spite of the high probability that costs for people without diabetes are likely to increase, they are kept constant in our analysis due to a lack of information on their precise development. The inclusion of doubtful cost trends might have impaired the trust in any cost estimate and its projection. Therefore, in line with Waldeyer et al. [7], we focused on an excess cost approach and limited the analysis to changes in costs of people with diabetes.

Lastly, it might seem critical that for simplicity and due to data unavailability, we assumed the same prevalence for German inhabitants and migrants. Though, Waldeyer et al. [7] showed that the impact of migration is negligible even when varying the prevalence.

Despite limitations, our study provides novel insights into the current and future economic burden of the two main types of diabetes in Germany. Although, type 1 and type 2 diabetes largely differ in their causes, symptoms, treatment and costs [23], previous studies rarely distinguished the types of diabetes [24]. For the first time, we projected type-specific direct medical cost, cost ratios and attributable costs related to the two main types of diabetes in Germany from 2010 until 2040 for both sex and all ages between 0 and 100 years based representative national routine data. Another advantage is the use of our forecasting model. Our method is able to reflect on temporal trends in epidemiological, demographic and cost dynamics simultaneously, but at the same time, remains transparent, clear and understandable in its application. Although our data and some assumptions may not be transferable, the statistical methods can be easily applied to other countries and chronic diseases and are flexible enough to anticipate impacts of alternative policy scenarios.

### Comparison with previous studies

Worldwide, studies of diabetes-related health expenditure report large disparities [25]. The few existing studies of diabetes-related costs in Germany are consistent in estimating a large economic burden, but results are heterogeneous [7, 14]. For our base year 2010 in Germany, studies reported diabetes-related per capita costs of €2,761 or €5,239 [22, 26]. Type-specific per capita healthcare costs associated with type 2 diabetes were estimated at €3,352, €4,377 and €5,146 [14, 27, 28]. Current per capita cost estimates issued by the International Diabetes Federation (IDF) amount to $6,661 for Germany in 2021 [25]. The studies differ largely with regards to their statistical methods, the cost components included, the inclusion criteria and encoding for diabetes diagnosis, the type of diabetes considered and the representativeness of their study population which renders comparison complicated if not impossible.

With regard to population attributable costs, two studies showed similar findings. In the CoDIM study, Köster et al. [22] used 2009 SHI data and estimated that diabetes patients incurred at least €21 billion more additional costs than people without diabetes. Jacobs et al. [14] estimated the costs solely related to type 2 diabetes in 2010 at €16.1 billion, which is slightly lower. For comparison, our analysis showed that in Germany in 2010, SHI expenses amounted to ~€160 billion. On average, 3.8% and 10.2% thereof are attributable to the medical care of type 1 and type 2 diabetes, respectively, which corresponds to about €6 and €16 billion. Our estimate is in line with the findings of Jacobs et al. [14] and only slightly exceeds the estimate provided by Köster et al. [22] (€22 billion vs. €21 billion). On the basis of these findings, we are confident that our results seem plausible.

For Germany, only two forecasts of diabetes-related costs are available [7]. One reports total annual costs of $30–56 billion in 2030 for type 1 and 2 of diabetes [25]. Due to lacking country-specific input data, an imbalanced ratio of individuals with versus without diabetes, the assumption of constant prevalence over time and the use of oversimplified methods, results may be overestimated. Using a time-discrete Markov model with locally limited data from the KORA (Cooperative Health Research in the Region of Augsburg) survey, Waldeyer et al. [7] projected total annual excess costs attributable to type 2 diabetes of €14,93 to €29.01 billion in 2040, with a baseline scenario yielding about €21 billion. These findings are comparable to our baseline scenario, projecting excess costs of almost €22 billion for type 2 diabetes in 2040.

### Implications

We found that the incidence and cost growth rate majorly drive future healthcare costs. This highlights the importance of population-based prevention and the need for supporting investments and cost policies, for instance in health infrastructure, new medications and technologies such as e-health for increased cost-efficiency.

In Germany, type 2 diabetes accounts for about 95% of all diabetes mellitus cases and in contrast to type 1 diabetes, can be prevented at little expense in the everyday life through cost effective and structured interventions [29]. To reduce the projected diabetes epidemic and its associated costs, the government, healthcare providers and institutions should put efforts to raise awareness and to intervene to prevent the onset of type 2 diabetes. This can be achieved through changing behaviour, norms and structures in order to support healthy living. Although these recommendations include upfront costs to the German government and healthcare system, we feel these investments are necessary.

## Conclusion

In summary, type 1 and 2 diabetes pose a notable economic burden to the individuals suffering from the disease, as well as to the German healthcare system, its economy and with that, the whole German population. Projected healthcare costs are highly sensitive to variation in cost growth and the incidence. Our analysis shows that by 2040, diabetes-related costs will probably increase substantially. Economic growth in Germany and subsequent increases in SHI budgets may compensate part of the increase. However, this would require promising developments in the German economy and well-planned financing by the government. To enable this, better and more timely epidemiological and economic diabetes-related data are needed in order to improve forecasting, to advance efforts at public awareness, and to support effective management and coordinated action for preventing and preparing for this development.

### Electronic supplementary material

Below is the link to the electronic supplementary material.

Supplementary Material 1

## Data Availability

The statistical analysis was carried out using the free statistical software R, version 4.1.2 (R Foundation for Statistical Computing). The source code and most of the data for running the analysis of the current study are published in the open-access repository Zenodo [17]. Due to regulations on data protection, routine SHI data were only available for our study and were provided in an anonymous and aggregated form (§ 5 Data Transparency Regulation, paragraph 4). Therefore, these data are not publicly available. An ethics committee approval was not required since no individual data on humans or animals were involved.
