# Supplementary material for: Projecting the economic burden of type 1 and type 2 diabetes mellitus in Germany from 2010 until 2040
Source: Popul Health Metr. 2024 Jul 18;22:17. doi: 10.1186/s12963-024-00337-x (PMC11264726; doi:10.1186/s12963-024-00337-x)
Supplement: Supplementary file 1 — Supplementary Material 1 [file 12963_2024_337_MOESM1_ESM.docx]

# Additional file 1

# Projecting the economic burden of type 1 and type 2 diabetes mellitus in Germany from 2010 until 2040

Dina Voeltz, M.Sc. (dina.voeltz@uni-bielefeld.de)^1,2^, Maximilian Vetterer, B.Sc. (mvetterer@googlemail.com)^2^, Dr. Esther Seidel-Jacobs (esther.seidel-jacobs@ddz.de) ^3,4^, Prof. Dr. Ralph Brinks (ralph.brinks@uni-wh.de)^3,5^, Dr. Thaddäus Tönnies (thaddaeus.toennies@ddz.de)^3,4^, Prof. Dr. Annika Hoyer (annika.hoyer@uni-bielefeld.de)^1^

^1^ Biostatistics and Medical Biometry, Medical School OWL, Bielefeld University, Bielefeld

^2^ Department of Statistics, Ludwig-Maximilians-University Munich

^3^ Institute for Biometrics and Epidemiology, German Diabetes Center, Leibniz Institute for Diabetes
 Research at Heinrich-Heine-University Düsseldorf

^4^ German Center for Diabetes Research, Partner Düsseldorf, München-Neuherberg, Germany

^5^ Chair for Medical Biometry and Epidemiology, Witten/Herdecke University, Faculty of Health/School of Medicine, Witten

## Overview of the input data, variables and primary outcomes

Additional Table 1: Overview of input data, variables and outcome measures

| *Variable* | *Definition* | *Source* | *Input value/ Derivation* |
| --- | --- | --- | --- |
| *N* | Population number,  year-, age- and sex-specific | 15^th^ Population Projection,  Federal Statistical Office of Germany [1, 2] | Variants G2L2W1, G2L2W3, G1L2W2, G1L3W1, G3L1W3, G2L2W2 |
| *p* | Prevalence,  year-, age- and sex-specific | *For type 1 diabetes:*  German Institute of Medial Documentation and Information, Tamayo et al. [3] & Voeltz et al. [4]  *For type 2 diabetes:*  German Institute of Medial Documentation and Information, Tamayo et al. [3], Tönnies et al. [5] & Voeltz et al. [6] | Type 1 diabetes prevalence in Germany in 2010  Type 2 diabetes prevalence in Germany in 2010  Prevalence projection until 2040 based on:  $\partial p=\left( 1-p \right)\times[IR- \frac{p\times(MRR-1)\times m}{p\times\left( MRR-1 \right)+1} ]$ |
| *IR* | Incidence rate,  year-, age-, sex- and type-specific | *See p* | $IR=\left( \frac{\partial}{\partial t}+\frac{\partial}{\partial a} \right)p/(1-p)+m \times\frac{p(MRR-1)}{1+p(MRR-1)}$ |
| *MRR* | Mortality rate ratio,  year-, age-, sex- and type-specific | *For type 1 diabetes:*  Carstensen et al. [7, 8] & Voeltz et al. [4]  *For type 2 diabetes:*  Schmidt et al. [9] & Voeltz et al. [6] | Type 1 diabetes-related MRR in Denmark in 1996 until 2017  Type 2 diabetes-related MRR in Germany in 2013 and 2014  $MRR = \frac{{mortality}_{diabetes}}{{mortality}_{nodiabetes}}$ |
| *m* | General mortality of the whole population,  year-, age- and sex-specific | 15^th^ Population Projection,  Federal Statistical Office of Germany [1, 2] | Variants G2L2W1, G2L2W3, G1L2W2, G1L3W1, G3L1W3, G2L2W2 |
| *cap* | Per capita costs,  year-, sex- and type-specific, categorized into 5-year age groups | Aggregated data from 4.3 million people (n) in 2009 and 2010 in Germany incl. ICD-10 coding from cost-of-illness-study based on a 6.8% random sample of all German statutory health insurances which we linearly interpolated between the age classes,  German Institute of Medial Documentation and Information, Tamayo et al. [3] & Jacobs et al. [10] |  |
| *n* | Number of individuals included in the cost-of-illness-study (sample size) | *See cap* |  |
| *C* | Total costs,  year-, sex- and type-specific | *See cap* | $C = n x cap$ |
| *K* | Average total costs,  year-, sex- and type-specific | *See cap* | $K=\sum C$ |
| *kap* | Average per capita costs,  year-, age-, sex- and type-specific | *See cap* | $kap = \frac{K}{n}$ |
| *E* | Excess per capita costs,  year-, age-, sex- and type-specific | *See cap* | $E = {kap}_{diabetes}-{kap}_{nodiabetes}$ |
| *R* | Cost ratio,  year-, age-, sex- and type-specific  Ratio of costs for people with type 1 or type 2 diabetes relative to people without | *See cap* | $R = \frac{{kap}_{diabetes}}{{kap}_{nodiabetes}}$ |
| *PAC* | Population attributable costs,  year-, age-, sex- and type-specific | *See cap* | $PAC= \frac{p\times(R-1)}{1+p\times(R-1)}$ |
| *Indices* |  |  |  |
| *d* | Diabetes status based on ICD-10 code | Diagnosed type 1 (ICD E10), diagnosed type 2 diabetes (ICD E11) or no diagnosed type 1 or 2 diabetes (no ICD coding E10-E14) |  |
| *a* | Age | All ages from 0 to 100 |  |
| *s* | Sex | Men or women |  |
| *t* | Year | All years from 2010 to 2040 |  |

## Derivation of the scenarios

Being limited by the availability of data on future epidemiological, demographic and cost trends, we constructed several speculative time-related scenarios that are motivated by previous projections of diabetes in Germany (see Additional Table 2). For comparison, we include one base case scenario (scenario 1) in which we apply only minimal changes over time to our input information. This scenario assumes moderate demographic developments (based on population projection variant G2L2W2) and is limited to an annual 2% decrease in the MRR. No changes are made to any other cost or epidemiological input.

Due to data scarcity regarding epidemiological future trends in Germany, we assessed several scenarios (scenarios 2 to 8) to show the impact of the incidence, mortality and prevalence on the sensitivity of our results. Our PDE allows us to reflect on temporal changes in disease-specific rates, as it models the prevalence as a function of incidence and mortality. Current evidence suggests that the mortality rate among people with diabetes is likely to decrease faster in the future than among people without diabetes due to developments in medical care [5, 7, 11]. Consequently, we consider a reduction in the MRR of 2% per year for all scenarios except scenario 8. In their projection of type 2 diabetes cases in Germany, Tönnies et al. [5] found that changes in the MRR had a minor (if not negligible) impact on the number of future cases. However, variation in the incidence rates had a strong effect on the number of future cases: “*A relatively low decrease in the incidence of −0.5% per year reduces the increase in future cases by 11 percentage points, compared with the scenario of constant incidence*”. We refrained from modelling additional changes in the MRR in order to not making the analysis needlessly complicated and complex. In scenarios 2 to 5, we additionally alter the incidence by annually -0.1%, -0.5%, +0.1% and 0.5%, respectively. With regards to epidemiological future trends, scenarios 1 to 5 are rather realistic compared to scenarios 6 and 7, which are based on an annual 5% increase or decrease of the incidence. As rationale for these extreme scenarios, Voeltz et al. [4, 6] point towards a reported 5% decrease of the incidence which was observed among older ages in Denmark between 1996 and 2016. Vice versa, they also reflect on an opposed 5% increase in the incidence, as for instance, researchers postulated that infections with the SARS-CoV-2 virus might notably increase the risk of diabetes due to cell damages. Lastly, scenario 8 assumes a constant age-specific prevalence throughout the entire projection horizon and neither reflects on the incidence nor on the mortality. It is based on a very profound and simple prevalence extrapolation, where the sex- and age-specific prevalence from the base year 2010 is combined with the population projections of the FSO. Although this method is not recommended and discussed as inaccurate due to its simplicity, we incorporate it as it is a common “status quo approach” in many epidemiological projection contexts.

To deal with the lack of precise information on temporal trends of the healthcare costs for people with and without diabetes in Germany, scenario 9 and 10 assess the impact of two speculative trends. Since particularly information on the general cost development of people without diabetes is limited, we align with Waldeyer et al. [12] and model the associated annual direct medical excess costs (instead of total costs) of type 1 or type 2 diabetes, respectively, from the payer’s perspective. Evidence from the past shows that it is unlikely that costs will decrease in the future. Consequently, we assume that healthcare costs for people with diabetes will continue to increase. In scenario 9, the mean annual excess costs inflation was predicted to increase by 1% until 2040. In this scenario, the mean annual growth rate of 1 % is constructed as dynamic cost increase as follows: Aligned with Waldeyer et al. [12], the cost increase was set to 5.0% annually until 2015, to diminish to 3.1% per year from 2015 to 2020 and to flatten to 1.9% from 2020 to 2025. After 2025, the growth in the excess costs was set to 0.966%. The rationale for considering a high increase of costs at first and then a slower cost growth in the far future could be as follows. Due to the rising relevance of diabetes, it is likely that investments will be made into research and development of new medications, drugs and therapies for its treatment. Obviously, these new medications are relatively expensive at first, leading to higher costs for the treatment of diabetes. After some years though, when the patent has expired, other pharmaceutical companies may follow and market very similar and cheaper drugs, so-called generics and biosimilars. In addition to scenario 9, and as a relatively extreme setting, scenario 10 is based on a constant increase of the excess costs of 5% per year.

Lastly, to assess the variability of our projected cost results with regards to demographic developments, scenario 12 to 15 account for potential trends in migration, life expectancy and birth rates of future Germany. For this aim, we consider different population projection variants issued by the FSO (see Additional Table 2).

Additional Table 2: Overview of our projection scenarios

| **Scenario** | **IR** | **MRR** | **Excess cost** | **FSO variant** | **Explanation** |
| --- | --- | --- | --- | --- | --- |
| 1 | 0% | -2% | 0% | G2L2W2 | Baseline scenario |
| 2 | -0.1% | -2% | 0% | G2L2W2 | Moderate annual decrease of the incidence |
| 3 | -0.5% | -2% | 0% | G2L2W2 | Moderate annual decrease of the incidence |
| 4 | 0.1% | -2% | 0% | G2L2W2 | Moderate annual increase of the incidence |
| 5 | 0.5% | -2% | 0% | G2L2W2 | Moderate annual increase of the incidence |
| 6 | 5% | -2% | 0% | G2L2W2 | Extreme epidemiological scenario |
| 7 | -5% | -2% | 0% | G2L2W2 | Extreme epidemiological scenario |
| 8 | constant | prevalence | 0% | G2L2W2 | Constant age-specific prevalence projection |
| 9 | 0% | -2% | 1% | G2L2W2 | Moderate annual excess cost inflation |
| 10 | 0% | -2% | 5% | G2L2W2 | Extreme cost scenario |
| 11 | 0% | -2% | 0% | G2L2W1 | Low annual migration rate |
| 12 | 0% | -2% | 0% | G2L2W3 | High annual migration rate |
| 13 | 0% | -2% | 0% | G1L2W2 | Decreasing birth rate |
| 14 | 0% | -2% | 0% | G1L3W1 | Relatively old population |
| 15 | 0% | -2% | 0% | G3L1W3 | Relatively young population |
| 16 | 0.1% | -2% | 1% | G2L2W2 | Most probable scenario |

## Justification of scenario 16 as “most likely”

With regard to the growth of costs related to diabetes treatment vs. overall medical costs, there is evidence that costs for the treatment of diabetes have largely increased, e.g. due to the increases in medications costs, increases in insulin cost and lack of generic glucose-lowering medication [13–16]. In a report of Heidemann et al. [17] published by the Robert Koch Institute (RKI) it is stated that according to Federal Statistical Office estimates the direct costs of caring for diabetes patients in Germany (outpatient and inpatient treatment, nursing, rehabilitation services and drugs) the direct costs of diabetes gradually increased over time. E.g., diabetes-related costs (excluding expenditures on the treatment of diabetic complications and secondary diseases) in 2008 were estimated 28% higher than in 2002, whereas costs of all other diseases had increased by only 16%. Direct costs related to diabetes including expenditures on the treatment of diabetic complications and secondary diseases are assumed about three times higher (for 2007: €19.1 billion, [18]). Further, if even indirect costs resulting from diabetes (e.g., loss of resources through disability or early retirement) would also be included, total costs could be as much as four times higher [19]. For our assumed cost development, we aligned with Waldeyer et al. [12] as this is the only comparable study that predicted the course of annual Type 2 diabetes-related direct medical costs in the German population from 2010 to 2040. To increase comparability of the projection of Waldeyer et al. [12] and our study, and due to scarce information on future temporal trends in the costs of people with and without diabetes, we adopted their assumptions on future cost developments.

We agree that the temporal trend of the incidence of type 1 and type 2 diabetes may not be the same in the future. However, literature provides heterogeneous temporal trends of the incidence of diabetes overall, of type 1 and type 2 diabetes. Estimates were largely dependent on the overall time horizon, the studied population, population size and representativeness of the sample for the whole German population. Thus, while some studies report increasing incidence, others report declining or stable rates.

For instance, a systematic review reporting type 2 diabetes incidence on a global level for the first time revealed increasing, stable and decreasing incidence trends for a high number of high- and middle-income countries since 2010. Precisely, Magliano et al. [20] found that in 1990 to 2005, diabetes incidence mostly increased in 66%, was stable in 32%, and decreased in 2% (1/50). Between 2006 to 14, increasing trends were reported in only 33%, whereas 30% had stable incidence and 36% declining incidence. Goffrier et al. [21] report that in Germany, the standardised prevalence of diabetes mellitus rose from 8.9% in 2009 to 9.8% in 2015. They state that this is primarily due to a rise in the prevalence of type 2 diabetes from 8.5% to 9.5%. They conclude that "based on predicted demographic change, it can be assumed that the disease burden due to diabetes mellitus will continue to rise".

In a very recent study from 2023, Reitzle et al. [22] showed the following: "*The incidence of type 1 diabetes increased from 9.5 to 11.6 per 100,000 people between 2015 and 2021 (from 7,007 to 8,699 new cases per year). The incidence of type 2 diabetes showed a decreasing trend between 2015 and 2019. During the pandemic, it initially fell further in 2020 and rose to 740 per 100,000 people in 2021 (556,318 new cases per year). During the pandemic years, the type-specific seasonal pattern of previous years was changed. For both type 1 and type 2 diabetes, a higher incidence was observed in regions with high than in regions with low socioeconomic deprivation. The increase in the incidence of type 1 and type 2 diabetes in 2021 may be related to the COVID-19 pandemic. The high incidences and the differences according to regional socioeconomic deprivation indicate a need for appropriate prevention strategies*."

Current studies support this finding and found evidence that Covid-19 may have led to an increased diabetes incidence [23–25]. It was hypothesized that either the COVID-19 infection itself or disease containment measures possibly altered the impact of lifestyle factors such as physical activity which ultimately increased the increase in type 2 diabetes [23, 25].

Overall, future surveillance is necessary to assess the actual development of diabetes incidence and influencing factors such as lifestyle change, the effect of the pandemic on diabetes incidence or screening activities. As noted in our article, we modelled several scenarios to assess and integrate uncertainty in our projection, but can only speculate which scenario is most likely. For our study, we concluded that it is more likely to find increasing trends in the future incidence. For ease of understanding, we assume similar trends for type 1 and type 2 diabetes. Further, due to scarcity of information and heterogeneity in previous, we aligned our epidemiological scenarios with previous, relatively recent projections of type 1 and type 2 diabetes in Germany [4–6] that covered the same time horizon.

## Additional visualisations of our projection results

**Additional Figure 1:** **Average annual per capita costs**

Projected age-specific per capita cost (in €) of people with type 1 or 2 diabetes in the statutory health insurance in Germany between 2010 and 2040 (stratified by sex). Panel a and b visualize average annual per capita costs for men and women with versus without type 1 diabetes, respectively. Panel c and d show results for men and women with versus without type 2 diabetes.


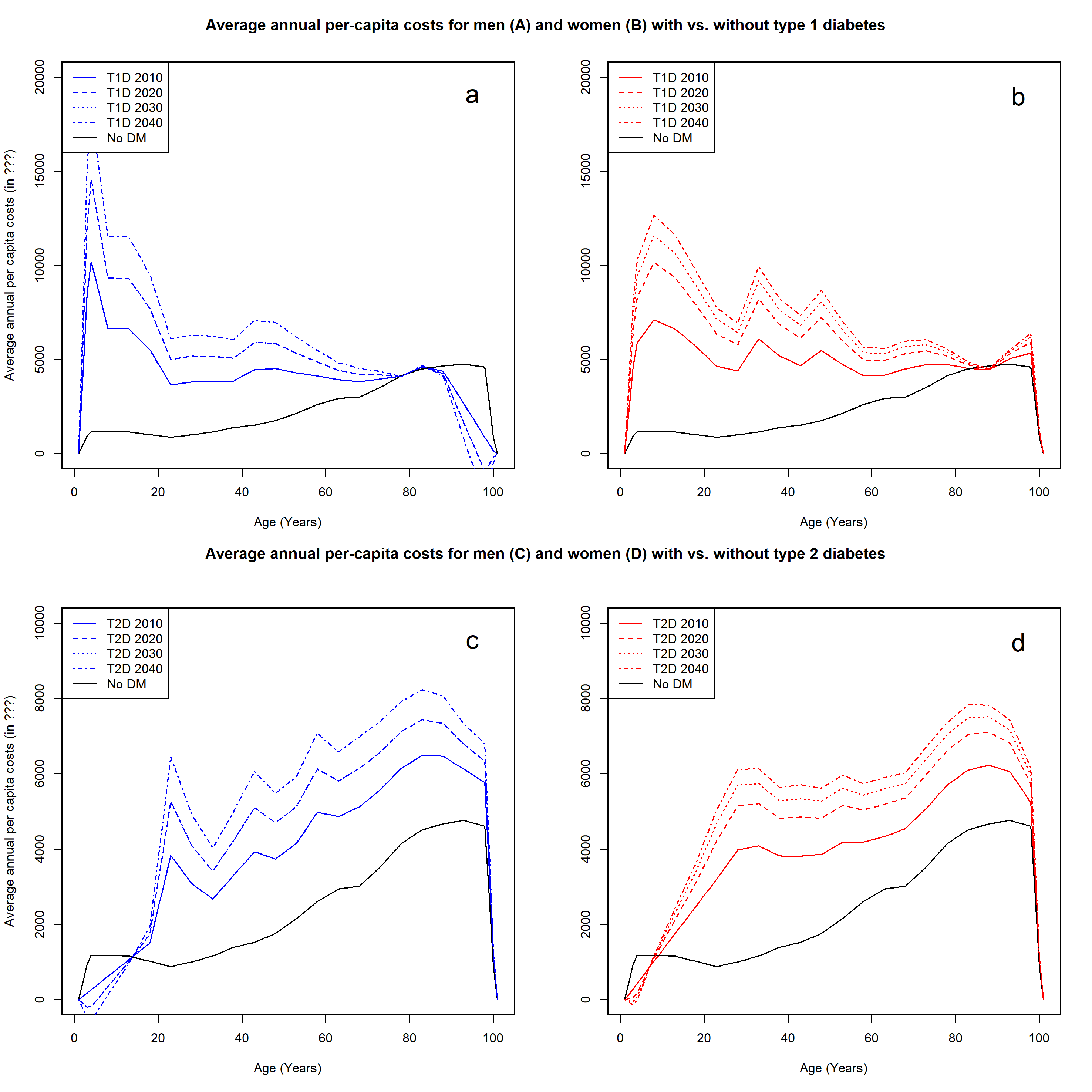


**Additional Figure 2: Projected cost ratios**

Age-specific (for ages 10, 20, 30, 50, 70, 90) cost ratios of the total healthcare expenses (in €) between men and women with diagnosed type 1 or 2 diabetes versus without in the statutory health insurance in Germany over time from 2010 to 2040. Cost ratios for type 1 diabetes are shown in panel a for men and b for women, cost ratios for type 2 diabetes are shown in panel c for men and d for women.


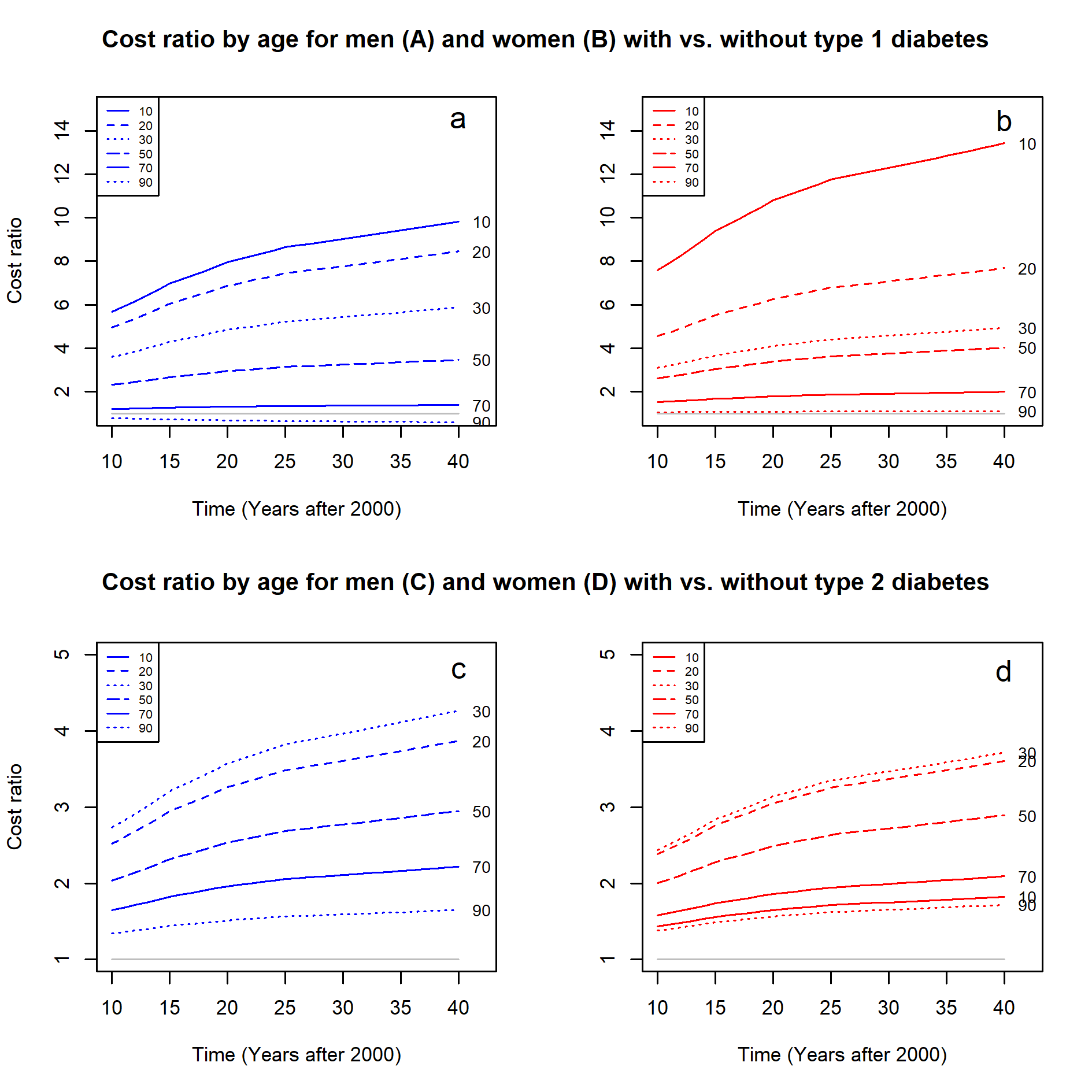


## Detailed results for different epidemiological-, demographical- and cost-scenarios

**Additional Table 3:** Annual projected total costs of type 1 diabetes in Germany from 2010 until 2040 by sex (in billions)

|  |  |  |  |  |  | Men |  |  |  |  |  | Women | |  |  |  |  |
| --- | --- | --- | --- | --- | --- | --- | --- | --- | --- | --- | --- | --- | --- | --- | --- | --- | --- |
| Scenario | MRR | IR | Prevalence | Excess costs | Population variant | 2010 | 2020 | 2030 | 2040 | Abs. change (2010 vs 2040) | Rel. change (2010 vs 2040) | 2010 | 2020 | 2030 | 2040 | Abs. change (2010 vs 2040) | Rel. change (2010 vs 2040) |
| 1 | 0.89 | 1 | NA | 1 | G2L2W2 | 0.59 | 0.60 | 0.66 | 0.72 | 0.13 | 0.22 | 0.55 | 0.58 | 0.70 | 0.79 | 0.25 | 0.45 |
| 2 | 0.89 | 0.99 | NA | 1 | G2L2W2 | 0.59 | 0.60 | 0.66 | 0.72 | 0.12 | 0.2 | 0.55 | 0.58 | 0.69 | 0.79 | 0.24 | 0.43 |
| 3 | 0.89 | 0.95 | NA | 1 | G2L2W2 | 0.59 | 0.60 | 0.65 | 0.69 | 0.10 | 0.16 | 0.55 | 0.57 | 0.68 | 0.76 | 0.21 | 0.38 |
| 4 | 0.89 | 1.01 | NA | 1 | G2L2W2 | 0.59 | 0.60 | 0.66 | 0.73 | 0.14 | 0.23 | 0.55 | 0.58 | 0.70 | 0.80 | 0.25 | 0.46 |
| 5 | 0.89 | 1.05 | NA | 1 | G2L2W2 | 0.59 | 0.61 | 0.68 | 0.76 | 0.16 | 0.28 | 0.55 | 0.58 | 0.72 | 0.83 | 0.29 | 0.52 |
| 6 | 0.89 | 1.05 | NA | 1 | G2L2W2 | 0.59 | 0.65 | 0.89 | 1.32 | 0.72 | 1.22 | 0.55 | 0.63 | 0.95 | 1.46 | 0.91 | 1.66 |
| 7 | 0.89 | 0.95 | NA | 1 | G2L2W2 | 0.59 | 0.57 | 0.54 | 0.50 | -0.10 | -0.16 | 0.55 | 0.54 | 0.56 | 0.54 | -0.01 | -0.02 |
| 8 | NA | NA | 1 | 1 | G2L2W2 | 0.59 | 0.60 | 0.62 | 0.62 | 0.02 | 0.04 | 0.55 | 0.55 | 0.58 | 0.58 | 0.03 | 0.06 |
| 9 | 0.89 | 1 | NA | 1.01 | G2L2W2 | 0.59 | 0.76 | 0.91 | 1.05 | 0.46 | 0.77 | 0.55 | 0.74 | 0.99 | 1.19 | 0.64 | 1.17 |
| 10 | 0.89 | 1 | NA | 1.05 | G2L2W2 | 0.59 | 0.81 | 1.24 | 1.95 | 1.35 | 2.28 | 0.55 | 0.78 | 1.36 | 2.27 | 1.72 | 3.14 |
| 11 | 0.89 | 1 | NA | 1 | G2L2W1 | 0.59 | 0.60 | 0.65 | 0.70 | 0.10 | 0.17 | 0.55 | 0.58 | 0.68 | 0.76 | 0.21 | 0.39 |
| 12 | 0.89 | 1 | NA | 1 | G2L2W3 | 0.59 | 0.60 | 0.68 | 0.75 | 0.16 | 0.27 | 0.55 | 0.58 | 0.71 | 0.82 | 0.27 | 0.49 |
| 13 | 0.89 | 1 | NA | 1 | G1L2W2 | 0.59 | 0.60 | 0.66 | 0.72 | 0.12 | 0.21 | 0.55 | 0.58 | 0.70 | 0.79 | 0.24 | 0.44 |
| 14 | 0.89 | 1 | NA | 1 | G1L3W1 | 0.59 | 0.60 | 0.65 | 0.70 | 0.10 | 0.17 | 0.55 | 0.58 | 0.68 | 0.76 | 0.21 | 0.39 |
| 15 | 0.89 | 1 | NA | 1 | G3L1W3 | 0.59 | 0.60 | 0.67 | 0.75 | 0.16 | 0.27 | 0.55 | 0.58 | 0.71 | 0.82 | 0.27 | 0.49 |
| 16 | 0.89 | 1.01 | NA | 1.01 | G2L2W2 | 0.59 | 0.76 | 0.91 | 1.05 | 0.46 | 0.77 | 0.55 | 0.74 | 0.99 | 1.19 | 0.64 | 1.17 |

**Additional Table 4:** Annual projected total costs of type 2 diabetes in Germany from 2010 until 2040 by sex (in billions)

|  |  |  |  |  |  | Men |  |  |  |  |  | Women | |  |  |  |  |
| --- | --- | --- | --- | --- | --- | --- | --- | --- | --- | --- | --- | --- | --- | --- | --- | --- | --- |
| Scenario | MRR | IR | Prevalence | Excess costs | Population variant | 2010 | 2020 | 2030 | 2040 | Abs. change (2010 vs 2040) | Rel. change (2010 vs 2040) | 2010 | 2020 | 2030 | 2040 | Abs. change (2010 vs 2040) | Rel. change (2010 vs 2040) |
| 1 | 0.89 | 1 | NA | 1 | G2L2W2 | 14.7 | 22.7 | 27.9 | 31.8 | 17.09 | 1.16 | 14.0 | 20.1 | 24.6 | 28.4 | 14.44 | 1.03 |
| 2 | 0.89 | 0.99 | NA | 1 | G2L2W2 | 14.7 | 22.6 | 27.7 | 31.4 | 16.64 | 1.12 | 14.0 | 20.0 | 24.4 | 28.0 | 14.03 | 1.00 |
| 3 | 0.89 | 0.95 | NA | 1 | G2L2W2 | 14.7 | 22.4 | 26.8 | 29.7 | 14.92 | 1.01 | 14.0 | 19.8 | 23.6 | 26.4 | 12.44 | 0.89 |
| 4 | 0.89 | 1.01 | NA | 1 | G2L2W2 | 14.7 | 22.8 | 28.1 | 32.3 | 17.54 | 1.19 | 14.0 | 20.2 | 24.8 | 28.8 | 14.86 | 1.06 |
| 5 | 0.89 | 1.05 | NA | 1 | G2L2W2 | 14.7 | 23.0 | 29.0 | 34.2 | 19.42 | 1.31 | 14.0 | 20.4 | 25.6 | 30.6 | 16.60 | 1.18 |
| 6 | 0.89 | 1.05 | NA | 1 | G2L2W2 | 14.7 | 26.4 | 42.2 | 61.8 | 47.03 | 3.18 | 14.0 | 23.4 | 37.8 | 57.3 | 43.36 | 3.09 |
| 7 | 0.89 | 0.95 | NA | 1 | G2L2W2 | 14.7 | 19.8 | 19.0 | 16.1 | 1.38 | 0.09 | 14.0 | 17.5 | 16.6 | 14.2 | 0.21 | 0.01 |
| 8 | NA | NA | 1 | 1 | G2L2W2 | 14.7 | 16.9 | 18.1 | 19.2 | 4.45 | 0.30 | 14.0 | 15.4 | 16.4 | 17.7 | 3.71 | 0.26 |
| 9 | 0.89 | 1 | NA | 1.01 | G2L2W2 | 14.7 | 26.9 | 35.2 | 42.0 | 27.21 | 1.84 | 14.0 | 23.6 | 30.7 | 37.1 | 23.15 | 1.65 |
| 10 | 0.89 | 1 | NA | 1.05 | G2L2W2 | 14.7 | 28.1 | 44.9 | 69.8 | 55.00 | 3.72 | 14.0 | 24.6 | 38.8 | 61.0 | 47.04 | 3.36 |
| 11 | 0.89 | 1 | NA | 1 | G2L2W1 | 14.7 | 22.7 | 27.9 | 31.8 | 17.09 | 1.16 | 14.0 | 20.1 | 24.5 | 28.3 | 14.35 | 1.02 |
| 12 | 0.89 | 1 | NA | 1 | G2L2W3 | 14.7 | 22.7 | 27.8 | 31.8 | 17.07 | 1.15 | 14.0 | 20.1 | 24.6 | 28.5 | 14.52 | 1.04 |
| 13 | 0.89 | 1 | NA | 1 | G1L2W2 | 14.7 | 22.7 | 27.9 | 31.8 | 17.09 | 1.16 | 14.0 | 20.1 | 24.6 | 28.4 | 14.44 | 1.03 |
| 14 | 0.89 | 1 | NA | 1 | G1L3W1 | 14.7 | 22.7 | 28.1 | 32.5 | 17.78 | 1.20 | 14.0 | 20.1 | 24.7 | 28.9 | 14.96 | 1.07 |
| 15 | 0.89 | 1 | NA | 1 | G3L1W3 | 14.7 | 22.7 | 27.6 | 31.1 | 16.38 | 1.11 | 14.0 | 20.1 | 24.4 | 27.9 | 13.90 | 0.99 |
| 16 | 0.89 | 1.01 | NA | 1.01 | G2L2W2 | 14.7 | 26.9 | 35.2 | 42.0 | 27.21 | 1.84 | 14.0 | 23.6 | 30.7 | 37.1 | 23.15 | 1.65 |

**Additional Table 5:** Annual projected excess costs of type 1 diabetes in Germany from 2010 until 2040 by sex (in billions)

|  |  |  |  |  |  | Men |  |  |  | Women |  |  |  |
| --- | --- | --- | --- | --- | --- | --- | --- | --- | --- | --- | --- | --- | --- |
| Scenario | MRR | IR | Prevalence | Excess costs | Population variant | 2010 | 2020 | 2030 | 2040 | 2010 | 2020 | 2030 | 2040 |
| 1 | 0.89 | 1 | NA | 1 | G2L2W2 | 0.30 | 0.32 | 0.35 | 0.37 | 0.29 | 0.33 | 0.40 | 0.44 |
| 2 | 0.89 | 0.99 | NA | 1 | G2L2W2 | 0.30 | 0.32 | 0.35 | 0.36 | 0.29 | 0.33 | 0.40 | 0.44 |
| 3 | 0.89 | 0.95 | NA | 1 | G2L2W2 | 0.30 | 0.32 | 0.34 | 0.35 | 0.29 | 0.32 | 0.39 | 0.42 |
| 4 | 0.89 | 1.01 | NA | 1 | G2L2W2 | 0.30 | 0.32 | 0.35 | 0.37 | 0.29 | 0.33 | 0.40 | 0.45 |
| 5 | 0.89 | 1.05 | NA | 1 | G2L2W2 | 0.30 | 0.33 | 0.36 | 0.39 | 0.29 | 0.33 | 0.42 | 0.47 |
| 6 | 0.89 | 1.05 | NA | 1 | G2L2W2 | 0.30 | 0.36 | 0.51 | 0.77 | 0.29 | 0.36 | 0.57 | 0.89 |
| 7 | 0.89 | 0.95 | NA | 1 | G2L2W2 | 0.30 | 0.3 | 0.27 | 0.22 | 0.29 | 0.30 | 0.31 | 0.28 |
| 8 | NA | NA | 1 | 1 | G2L2W2 | 0.30 | 0.29 | 0.29 | 0.29 | 0.29 | 0.28 | 0.29 | 0.28 |
| 9 | 0.89 | 1 | NA | 1.01 | G2L2W2 | 0.30 | 0.48 | 0.60 | 0.70 | 0.29 | 0.49 | 0.69 | 0.84 |
| 10 | 0.89 | 1 | NA | 1.05 | G2L2W2 | 0.30 | 0.53 | 0.93 | 1.60 | 0.29 | 0.53 | 1.07 | 1.92 |
| 11 | 0.89 | 1 | NA | 1 | G2L2W1 | 0.30 | 0.32 | 0.34 | 0.35 | 0.29 | 0.33 | 0.39 | 0.42 |
| 12 | 0.89 | 1 | NA | 1 | G2L2W3 | 0.30 | 0.32 | 0.36 | 0.39 | 0.29 | 0.33 | 0.41 | 0.46 |
| 13 | 0.89 | 1 | NA | 1 | G1L2W2 | 0.30 | 0.32 | 0.35 | 0.37 | 0.29 | 0.33 | 0.40 | 0.44 |
| 14 | 0.89 | 1 | NA | 1 | G1L3W1 | 0.30 | 0.32 | 0.34 | 0.35 | 0.29 | 0.33 | 0.39 | 0.42 |
| 15 | 0.89 | 1 | NA | 1 | G3L1W3 | 0.30 | 0.32 | 0.36 | 0.39 | 0.29 | 0.33 | 0.41 | 0.46 |
| 16 | 0.89 | 1.01 | NA | 1.01 | G2L2W2 | 0.30 | 0.48 | 0.60 | 0.70 | 0.29 | 0.49 | 0.69 | 0.84 |

**Additional Table 6:** Annual projected excess costs of type 1 diabetes in Germany from 2010 until 2040 by sex (in billions)

|  |  |  |  |  |  | Men |  |  |  | Women |  |  |  |
| --- | --- | --- | --- | --- | --- | --- | --- | --- | --- | --- | --- | --- | --- |
| Scenario | MRR | IR | Prevalence | Excess costs | Population variant | 2010 | 2020 | 2030 | 2040 | 2010 | 2020 | 2030 | 2040 |
| 1 | 0.89 | 1 | NA | 1 | G2L2W2 | 5.76 | 8.56 | 10.27 | 11.41 | 5.03 | 7.15 | 8.62 | 9.81 |
| 2 | 0.89 | 0.99 | NA | 1 | G2L2W2 | 5.76 | 8.54 | 10.19 | 11.25 | 5.03 | 7.13 | 8.55 | 9.67 |
| 3 | 0.89 | 0.95 | NA | 1 | G2L2W2 | 5.76 | 8.44 | 9.85 | 10.6 | 5.03 | 7.04 | 8.26 | 9.10 |
| 4 | 0.89 | 1.01 | NA | 1 | G2L2W2 | 5.76 | 8.59 | 10.36 | 11.58 | 5.03 | 7.17 | 8.70 | 9.96 |
| 5 | 0.89 | 1.05 | NA | 1 | G2L2W2 | 5.76 | 8.70 | 10.72 | 12.29 | 5.03 | 7.26 | 9.00 | 10.59 |
| 6 | 0.89 | 1.05 | NA | 1 | G2L2W2 | 5.76 | 10.04 | 15.91 | 23.07 | 5.03 | 8.38 | 13.5 | 20.4 |
| 7 | 0.89 | 0.95 | NA | 1 | G2L2W2 | 5.76 | 7.42 | 6.87 | 5.58 | 5.03 | 6.20 | 5.75 | 4.78 |
| 8 | NA | NA | 1 | 1 | G2L2W2 | 5.76 | 6.45 | 6.82 | 7.08 | 5.03 | 5.49 | 5.80 | 6.16 |
| 9 | 0.89 | 1 | NA | 1.01 | G2L2W2 | 5.76 | 12.73 | 17.61 | 21.54 | 5.03 | 10.63 | 14.77 | 18.52 |
| 10 | 0.89 | 1 | NA | 1.05 | G2L2W2 | 5.76 | 13.95 | 27.26 | 49.33 | 5.03 | 11.64 | 22.87 | 42.41 |
| 11 | 0.89 | 1 | NA | 1 | G2L2W1 | 5.76 | 8.56 | 10.28 | 11.39 | 5.03 | 7.15 | 8.61 | 9.76 |
| 12 | 0.89 | 1 | NA | 1 | G2L2W3 | 5.76 | 8.56 | 10.26 | 11.43 | 5.03 | 7.15 | 8.63 | 9.86 |
| 13 | 0.89 | 1 | NA | 1 | G1L2W2 | 5.76 | 8.56 | 10.27 | 11.41 | 5.03 | 7.15 | 8.62 | 9.81 |
| 14 | 0.89 | 1 | NA | 1 | G1L3W1 | 5.76 | 8.56 | 10.35 | 11.60 | 5.03 | 7.15 | 8.67 | 9.94 |
| 15 | 0.89 | 1 | NA | 1 | G3L1W3 | 5.76 | 8.56 | 10.19 | 11.22 | 5.03 | 7.15 | 8.57 | 9.67 |
| 16 | 0.89 | 1.01 | NA | 1.01 | G2L2W2 | 5.76 | 12.73 | 17.61 | 21.54 | 5.03 | 10.63 | 14.77 | 18.52 |

References

1. Federal Statistical Office of Germany. 15. Coordinated population projection. 2023. https://www.destatis.de/EN/Themes/Society-Environment/Population/. Accessed 12 Apr 2023.

2. Federal Statistical Office of Germany. 14. Coordinated population projection. 2019. https://www.destatis.de/EN/Themes/Society-Environment/Population/. Accessed 12 Apr 2023.

3. Tamayo T, Brinks R, Hoyer A, Kuß O, Rathmann W. The prevalence and incidence of diabetes in Germany: an analysis of statutory health insurance data on 65 million individuals from the years 2009 and 2010. Deutsches Ärzteblatt International. 2016;113:177.

4. Voeltz D, Brinks R, Tönnies T, Hoyer A. Future number of people with diagnosed type 1 diabetes in Germany until 2040: an analysis based on claims data. BMJ Open Diabetes Research and Care. 2023;11:e003156.

5. Tönnies T, Röckl S, Hoyer A, Heidemann C, Baumert J, Du Y, Scheidt-Nave C, Brinks R. Projected number of people with diagnosed Type 2 diabetes in Germany in 2040. Diabetic Medicine. 2019;36:1217–25.

6. Voeltz D, Tönnies T, Brinks R, Hoyer A. Future prevalence of type 2 diabetes—A comparative analysis of chronic disease projection methods. Plos one. 2022;17:e0264739.

7. Carstensen B, Kristensen JK, Ottosen P, Borch-Johnsen K. The Danish National Diabetes Register: trends in incidence, prevalence and mortality. Diabetologia. 2008;51:2187–96.

8. Carstensen B, Rønn PF, Jørgensen ME. Prevalence, incidence and mortality of type 1 and type 2 diabetes in Denmark 1996‐2016. BMJ Open Diabetes Research and Care. 2020;8:e001071.

9. Schmidt C, Reitzle L, Heidemann C, Paprott R, Ziese T, Scheidt-Nave C, Baumert J. Excess mortality in adults with documented diabetes in Germany: routine data analysis of all insurance claims in Germany 2013‐2014. BMJ open. 2021;11:e041508.

10. Jacobs E, Hoyer A, Brinks R, Icks A, Kuß O, Rathmann W. Healthcare costs of Type 2 diabetes in Germany. Diabetic Medicine. 2017;34:855–61.

11. Gregg EW, Cheng YJ, Srinivasan M, Lin J, Geiss LS, Albright AL, Imperatore G. Trends in cause-specific mortality among adults with and without diagnosed diabetes in the USA: an epidemiological analysis of linked national survey and vital statistics data. The Lancet. 2018;391:2430–40.

12. Waldeyer R, Brinks R, Rathmann W, Giani G, Icks A. Projection of the burden of type 2 diabetes mellitus in Germany: a demographic modelling approach to estimate the direct medical excess costs from 2010 to 2040. Diabetic Medicine. 2013;30:999–1008.

13. Gao CC, Espinoza Suarez NR, Toloza FJK, Malaga Zuniga AS, McCarthy SR, Boehmer KR, et al. Patients' Perspective About the Cost of Diabetes Management: An Analysis of Online Health Communities. Mayo Clin Proc Innov Qual Outcomes. 2021;5:898–906. doi:10.1016/j.mayocpiqo.2021.07.003.

14. Hua X, Carvalho N, Tew M, Huang ES, Herman WH, Clarke P. Expenditures and prices of antihyperglycemic medications in the United States: 2002-2013. Jama. 2016;315:1400–2.

15. Gabler M, Picker N, Geier S, Foersch J, Aberle J, Martin S, et al. Real-world clinical outcomes and costs in type 2 diabetes mellitus patients after initiation of insulin therapy: a German claims data analysis. Diabetes research and clinical practice. 2021;174:108734.

16. Parker ED, Lin J, Mahoney T, Ume N, Yang G, Gabbay RA, et al. Economic Costs of Diabetes in the U.S. in 2022. Diabetes care. 2024;47:26–43. doi:10.2337/dci23-0085.

17. Heidemann C, Du Y, Schubert I, Rathmann W, Scheidt-Nave C. Prävalenz und zeitliche Entwicklung des bekannten Diabetes mellitus. Bundesgesundheitsblatt - Gesundheitsforschung - Gesundheitsschutz. 2013;56:668–77. doi:10.1007/s00103-012-1662-5.

18. Köster I, Huppertz E, Hauner H, Schubert I. Costs of Diabetes Mellitus (CoDiM) in Germany, direct per-capita costs of managing hyperglycaemia and diabetes complications in 2010 compared to 2001. Experimental and Clinical Endocrinology & Diabetes. 2014;122:510–6.

19. Hauner H. Die Kosten des Diabetes und seiner Komplikationen in Deutschland. DMW-Deutsche Medizinische Wochenschrift. 2006;131:S240‐S242.

20. Magliano DJ, Islam RM, Barr ELM, Gregg EW, Pavkov ME, Harding JL, et al. Trends in incidence of total or type 2 diabetes: systematic review. BMJ 2019. doi:10.1136/bmj.l5003.

21. Goffrier B, Schulz M, Bätzing-Feigenbaum J. Administrative Prevalence and Incidence of Diabetes Mellitus in Germany, 2009‐2015. In: Versorgungsatlas Report No. 17/03: Central Research Institute of Ambulatory Health Care in Germany (Zi) Berlin; 2017.

22. Reitzle L, Heidemann C, Jacob J, Pawlowska-Phelan D, Ludwig M, Scheidt-Nave C. Inzidenz von Typ-1-und Typ-2-Diabetes vor und während der COVID-19-Pandemie in Deutschland: Analyse von Routinedaten der Jahre 2015 bis 2021. 2023.

23. Marçal IR, Fernandes B, Viana AA, Ciolac EG. The urgent need for recommending physical activity for the management of diabetes during and beyond COVID-19 outbreak. Frontiers in Endocrinology. 2020;11:584642.

24. Lehner CT, Eberl M, Donnachie E, Tanaka LF, Schauberger G, Schederecker F, et al. Incidence trend of type 2 diabetes from 2012 to 2021 in Germany: an analysis of health claims data of 11 million statutorily insured people. Diabetologia. 2024;67:1040–50. doi:10.1007/s00125-024-06113-8.

25. Rathmann W, Kuss O, Kostev K. Incidence of newly diagnosed diabetes after Covid-19. Diabetologia. 2022;65:949–54. doi:10.1007/s00125-022-05670-0.
